# An Intensive Longitudinal Study of the Association of Stress With Hyperglycemia Using Real-Time Data Collection

**DOI:** 10.1093/geroni/igab046.680

**Published:** 2021-12-17

**Authors:** Brent Mausbach

**Affiliations:** University of California San Diego, La Jolla, California, United States

## Abstract

Caregivers of persons with dementia (PWD) are at significantly elevated risk for cardiovascular disease (CVD)s. A higher risk for diabetes is one potential mechanism of morbidity in caregivers. Diabetes has been associated with dyslipidemia, hypertension, oxidative stress, increased low-grade inflammation, and endothelial dysfunction, which all place individuals at risk for CVD. Elevated blood glucose, even in the nondiabetic range, is a significant risk marker for the development of CVD. The current study examined the semi-continuous association between stress and glucose. Participants wore a continuous glucose monitor that measured blood glucose every 5 minutes for a period of 10 days (n = 2,880/participant). Ecological Momentary Assessment (EMA) was used to measure stress, positive affect, negative affect, and dietary intake 3x/day over the 10-day period. Hierarchical linear models indicated significant within-person associations between stress and blood glucose levels (t = 3.88, df = 3.92, p = .018; R2 = 26.2%).

